# Adaptive Reconfiguration of Natural Killer Cells in HIV-1 Infection

**DOI:** 10.3389/fimmu.2018.00474

**Published:** 2018-03-16

**Authors:** Dimitra Peppa, Isabela Pedroza-Pacheco, Pierre Pellegrino, Ian Williams, Mala K. Maini, Persephone Borrow

**Affiliations:** ^1^Division of Infection and Immunity, University College London (UCL), London, United Kingdom; ^2^Nuffield Department of Clinical Medicine, University of Oxford, Oxford, United Kingdom; ^3^Centre for Sexual Health and HIV Research, University College London (UCL), London, United Kingdom

**Keywords:** natural killer cells, human cytomegalovirus, HIV-1, adaptive, PLZF

## Abstract

Human cytomegalovirus (HCMV) co-infection is highly prevalent within HIV-1 cohorts and is an important cofactor in driving ongoing immune activation, even during effective antiretroviral treatment. HCMV infection has recently been associated with expansion of adaptive-like natural killer (NK) cells, which harbor epigenetic alterations that impact on their cellular function and phenotype. The influence of HCMV co-infection on the considerable heterogeneity among NK cells and their functional responses to different stimuli was assessed in a cohort of HIV-1-infected individuals sampled during different stages of infection, compared with healthy subjects stratified according to HCMV serostatus. Our data demonstrate a reshaping of the NK cell pool in HIV-1 infection of HCMV-seropositive individuals, with an accentuated peripheral transition of CD56dim NK cells toward a mature CD57+ CD85j+ NKG2C+ NKG2A− phenotype. Lack of PLZF further distinguishes adaptive NK cells from other NK cells expressing CD57 or NKG2C. PLZF− NK cells from HIV-infected individuals had high expression of CD2, were Siglec-7 negative and exhibited downregulation of key signaling molecules, SYK and FcεRI-γ, overwhelmingly displaying features of adaptive NK cells that correlated with HCMV serum Ab levels. Notably this adaptive-like signature was detected during early HIV-1 infection and persisted during treatment. Adaptive-like NK cell subsets in HIV-1-infected individuals displayed enhanced IFN-γ production following Fc receptor triggering compared with their conventional NK cell counterparts, and their ability to produce TNF-α and degranulate was preserved. Together, these data suggest that HMCV infection/reactivation, a hallmark of HIV-1 infection, plays a role in driving a relative expansion of NK cells with adaptive features during HIV-1 infection. The identification of selective NK subsets with retained effector activity in HIV-1-infected subjects raises the possibility of developing therapeutic strategies that exploit specific NK subpopulations to achieve better HIV-1 control.

## Introduction

Natural killer (NK) cells are a diverse subset of innate lymphocytes that play a key effector role in antiviral defense, especially against herpesviruses (1), and tumor surveillance (2), and also have important immunoregulatory functions (3). In animal models, NK cells have been shown to have the capacity to acquire immunological memory, a property traditionally ascribed to B and T cells (4, 5).

A role for NK cells in HIV-1 infection is supported by epidemiological studies linking specific KIR/HLA combinations with HIV-1 infection outcome (6, 7), functional studies where protective KIR alleles are associated with enhanced NK cell cytolytic function *in vitro* (8) and evidence of HIV-1 having evolved strategies to evade NK cell recognition (9). In addition to genetic contributions influencing the NK cell repertoire environmental factors, especially infections, exert a profound and cumulative influence shaping NK cell diversity (10). Recent studies have shown that NK cells responding to murine CMV expand, forming a pool of long-lived memory cells that undergo robust recall responses (11). Human cytomegalovirus (HCMV) infection has also been linked with the identification of adaptive or memory-like NK cells in humans. These lasting expansions were originally characterized by higher frequencies of NKG2C+ NK cells in HCMV-seropositive individuals and/or in the context of acute HCMV infection or reactivation (12, 13). Such expansions have also been reported during acute and chronic viral infections including HIV-1, systematically associated with HCMV seropositivity (14). HCMV-adapted NK cells encompass heterogeneous populations characterized by a number of phenotypic attributes, not necessarily combined at a single-cell level or limited to the expression of NKG2C (15, 16). A degree of redundancy is evidenced by the detection of NK cell subsets sharing numerous phenotypic and functional attributes of adaptive NK cells in individuals independent of NKG2C or in the absence of NKG2C (*KLRC2*-deficient individuals) and in transplant recipients of NKG2C null grafts (16–18). Importantly, these so-called adaptive NK cells share epigenetic similarities to cytotoxic CD8 T cells and are marked by DNA methylation-dependent silencing of the transcription factor promyelocytic leukemia zinc (PLZF), as well as stochastic loss of expression of key proximal signaling molecules such as FcεRI-γ, spleen tyrosine kinase (SYK), or EWS/FLI1-activated transcript 2 (15, 19, 20). Adaptive NK cells are functionally dichotomized, exhibiting reduced responsiveness to stimulation with IL-12/IL-18 and a bias toward Fc receptor-dependent functions and specialization in the immunosurveillance of infected cells, and may be important for HCMV control especially when T cell immunity is impaired (21).

A comprehensive analysis of the transcriptional signatures and epigenetic modifications of NK cells in HIV-1 infection is currently lacking. Previous studies addressing the effects of HIV-1 infection on NK cell phenotypic and functional attributes do not always account for the confounding effect of HCMV, which is almost universal in HIV-1-infected cohorts and is an important cofactor associated with systemic inflammation, non-AIDS complications and immune senescence (22, 23), as well as an important driver of NK cell-associated changes (24). Here, we explored adaptive-like NK expansions during the course of HIV-1 infection, taking into account the likely influence of co-infection with HCMV. We also characterized NK effector responsiveness to various stimuli to identify novel NK cell “adaptive” signatures and preserved activation pathways that could be targeted for therapeutic purposes. Our data indicate a reshaping of the NK cell CD56dim pool bearing the trademarks of HCMV co-infection.

## Materials and Methods

### Study Subjects

For cross-sectional analyses, PBMCs cryopreserved from 21 untreated chronically HIV-1-infected Caucasian males from a London cohort were utilized. The London cohort was recruited at the Mortimer Market Center for Sexual Health and HIV Research (London, UK) following written informed consent as part of a study approved by the local ethics board committee. The cross-sectionally studied individuals formed part of a historical cohort who presented with symptoms of acute retroviral infection and were followed-up longitudinally into chronic infection and post commencement of antiretroviral treatment (ART). The date of infection was estimated as 17 days before symptom onset, which we assumed to occur in Fiebig stage 2, i.e., in the phase of acute HIV-1 infection before the peak in viremia when HIV-1 RNA and p24 antigen can be detected in plasma but seroconversion has not yet started to occur (25). A subgroup of five individuals from whom early infection samples was also available, was used for longitudinal assessment of the NK cell repertoire from early to chronic to posttreatment time points. At the post-treatment time point, all subjects had been receiving ART continually for at least 12 months and had levels of viremia <50 RNA copies/mL. Twenty demographically age- and sex-matched HIV-1 seronegative controls were used for comparison, from whom blood was taken and cryopreserved for later use with written informed consent in accordance with the Declaration of Helsinki. All study participants were anti-Hepatitis C virus antibody negative and anti HBsAg negative. HCMV serology was determined at University College London Hospital clinical virology lab by the ARCHITECT CMV IgG assay (AU/mL) (Abbott Diagnostics, IL, USA). Patient and healthy control characteristics are included in Tables S1 and S2 in Supplementary Material.

### Monoclonal Antibodies and Flow Cytometric Analysis

The following fluorochrome-conjugated antibodies were used in this study: CD14 BV510, CD19 BV510, CD56 PE Dazzle, CD3 BV650, CD16 PERCP or CD16 BV711, CD38 BV785, NKG2D FITC, PD1 BV711 (BioLegend), NKp30 APC (Miltenyi), NKp46 PE (BD Biosciences), CRACC Pe-Cy7, CD2 Pe-Cy7, Siglec-7 PE, 2B4 APC (BioLegend), CD85j biotin (eBioscience) followed by Streptavidin-BV711 (BioLegend), CD4 APC-Cy7, CD8 Alexa700 (eBioscience), NKG2A Pe-Cy7, KIR2DL2 APC CD158b1/b2.j APC (Beckman Coulter), NKG2C PE, KIR2DL1/2DS5 APC IgG1 (CD158a), KIR3DL2 APC (R&D Systems), CD57 BV421 (BD Biosciences), KIR3DL1 APC (CD158e1) (Miltenyi), for surface antigens; CD3ζ, Syk PE (BD Biosciences), Perforin PE (BioLegend), IFN-γ BV421 (BD Biosciences), TNF-α BV711 (BioLegend), FcεRI-γ-FITC (Millipore) for intracellular staining; and PLZF-APC (BD Biosciences) for intranuclear staining. Briefly, cryopreserved PBMC isolated from HIV-1-infected patients and healthy donors were washed in PBS, and surface stained at 4°C for 20 min with saturating concentrations of different combinations of antibodies in the presence of fixable live/dead stain (Invitrogen). Cells were then fixed and permeabilized for detection of intracellular antigens. The Foxp3 intranuclear staining buffer kit (eBioscience) was used according to the manufacturer’s instructions for the detection of intranuclear markers. Samples were acquired on a BD Fortessa X20 using BD FACSDiva8.0 (BD Bioscience) and data analyzed using FlowJo 10 (TreeStar). Stochastic neighbor embedding (SNE) analysis was performed using the mrc.cytobank platform. The t-distributed stochastic neighbor embedding algorithm clusters cells according to expression of multiple parameters and enables visualization of high-dimensional data in two-dimensional representations (single-cell spatial groupings), avoiding the bias that can be introduced by manual gating of specific subsets (26). For concatenating multiple FCS files into a single FCS file, the FCS file concatenation tool was used before uploading the files to cytobank. NK cells were identified based on CD3, CD56 and CD16 expression. For the panel of differentiation markers the viSNE calculation was based on the parameters PLZF, FcεRI-γ, CD2, and Siglec-7.

### Functional Assays

For target cell stimulation PBMC were incubated with K562 cells (5:1 E:T ratio) for 6 h at 37°C in the presence of CD107a-APC-Cy7 antibody (BD Biosciences, Cowley, UK) and GolgiStop (containing Monensin, 1/1,500 concentration, BD Biosciences). To assess responses to stimulation with cytokines, PBMC were incubated with 10 ng/mL of rhIL-12 (PeproTech) and 100 ng/mL rhIL-18 (R&D Systems, Abingdon, UK) for 18 h at 37°C. GolgiStop (containing Monensin, 1/1,500 concentration, BD Biosciences) and GolgiPlug (containing brefeldin A, 1/1,000 final concentration, BD Biosciences) were added for the last 3 h of culture in experiments where intracellular IFN-γ was a read-out. Where indicated stimulation of PBMC with phorbol 12-myristate 13-acetate (PMA) (3 ng/mL), and ionomycin (100 ng/mL) (Sigma) was performed for 6 h.

For activation *via* CD16 cross-linking, 96-well flat-bottom plates (Nunc) were coated with 5 μg/ml antihuman CD16 (clone 3G8, BD Biosciences) or an isotype-matched control antibody (mIgG1κ, BD Biosciences) overnight at 4°C. Plates were washed with sterile PBS before addition of 4 × 10^5^ PBMC per well. Cells were incubated for 6 hrs in the presence of CD107a-APC-Cy7 antibody (BD Biosciences, Cowley, UK). GolgiStop (containing Monensin, 1/1,500 concentration, BD Biosciences) and GolgiPlug (containing brefeldin A, 1/1,000 final concentration, BD Biosciences) were added for the last 5 h of culture. Following incubation cells were washed and stained for extracellular receptors before permeabilization and intracellular staining for TNF-α and IFN-γ.

### DNA Methylation Analysis

Genomic DNA was isolated using the DNeasy Blood and Tissue kit (QIAGEN). The methylation levels of seven CPG residues within the *IFNG* CNS1 region were analyzed *via* bisulfite conversion and pyrosequencing by Epigendx, Inc. The Human *IFNG* methylation assay ADS2902-FS1 (−4,394 to −4,355 from TSS) and ADS2902-FS2 (−4,320 to −4,224 from TSS) distal promoter (CNS1) were used. Donors were selected based on the size of the target subsets to ensure sufficient numbers of cells for methylation analysis after sorting.

### Data Analysis

Prism 7 (GraphPad Software) was used for all statistical analysis as follows: the Mann–Whitney *U*-test or Student’s *t*-test were used for single comparisons of independent groups, the Wilcoxon-test was used to compare two paired groups, Kruskal–Wallis with Dunn’s multiple comparison test was used to compare three unpaired sample groups, and Friedman test was used for multiple comparison of paired samples. The non-parametric Spearman test was used for correlation analysis. Permutation tests were performed in SPICE version 5.32 (*p < 0.05, **p < 0.01, ***p < 0.001, and ****p < 0.0001).

## Results

### Skewing of the NK Cell Repertoire toward a Mature/Terminally Differentiated Phenotype during Viremic HIV-1 Infection

**Figure 1:**
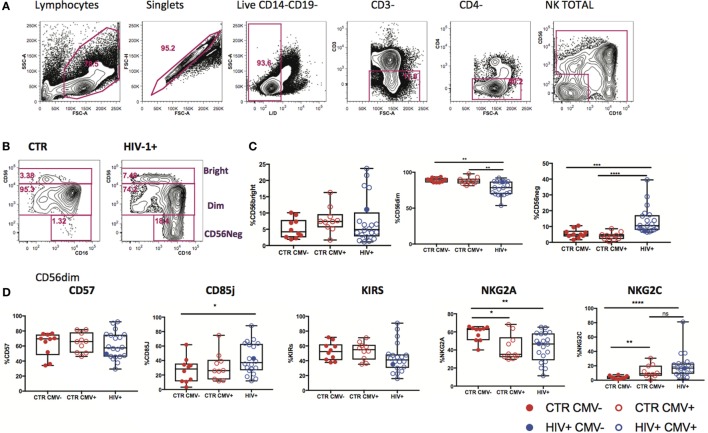
Subset redistribution and NK cell receptor (NKR) expression according to human cytomegalovirus (HCMV) serostatus. **(A)** Representative example gated on live CD3− CD4− lymphocytes; CD56 and CD16 are used to identify natural killer (NK) cells, discriminating between populations on the basis of CD56bright, dim, and negative expression levels. **(B)** Representative example of NK cell subset distribution in a healthy control versus a HIV-1-infected individual. **(C)** Summary box plots of the frequencies of CD56bright, CD56dim, and CD56neg NK cell subsets among HIV-1-uninfected HCMV− (CTR CMV−), HIV-1-uninfected HCMV+, and HIV-1-infected individuals. Red filled circles denote CTR CMV− subjects, open circles denote CTR CMV+, blue open circles denote HIV-1-infected HCMV+ individuals, and the single-filled blue dot denotes the HIV-1-infected HCMV− subject. **(D)** Summary data for the expression of NKRs as shown in the CD56dim subset for the three groups studied. Significance determined by the Mann–Whitney test for comparison between groups (*p < 0.05, **p < 0.01, and ****p < 0.0001).

Changes in expression of NK cell receptors (NKRs) compared with those in healthy volunteers have been reported in viremic HIV-1 infection (27, 28), but the HCMV serostatus of the subjects studied has not always been taken into consideration. We explored the diversity of NK cell phenotypes in a unique cohort of *n* = 21 HIV-1-infected male Caucasian individuals (*n* = 20 HCMV-seropositive, *n* = 1 HCMV seronegative) recruited during acute infection and followed-up longitudinally both pre- and post-initiation of antiretroviral therapy, and compared with two groups of *n* = 10 HCMV-seropositive and *n* = 10 HCMV seronegative HIV-1 seronegative age matched male Caucasian controls (Tables S1 and S2 in Supplementary Material). We confirmed the effects of HIV-1 viremia on the pathological redistribution of the NK cell compartment with a reduction in frequencies of the CD56dim subset and the emergence of an aberrant CD56−CD16+ NK cell subset as previously described (29, 30) compared with the HIV-1 seronegative controls and irrespective of their HCMV serostatus (Figures 1A–C).

**Figure 2:**
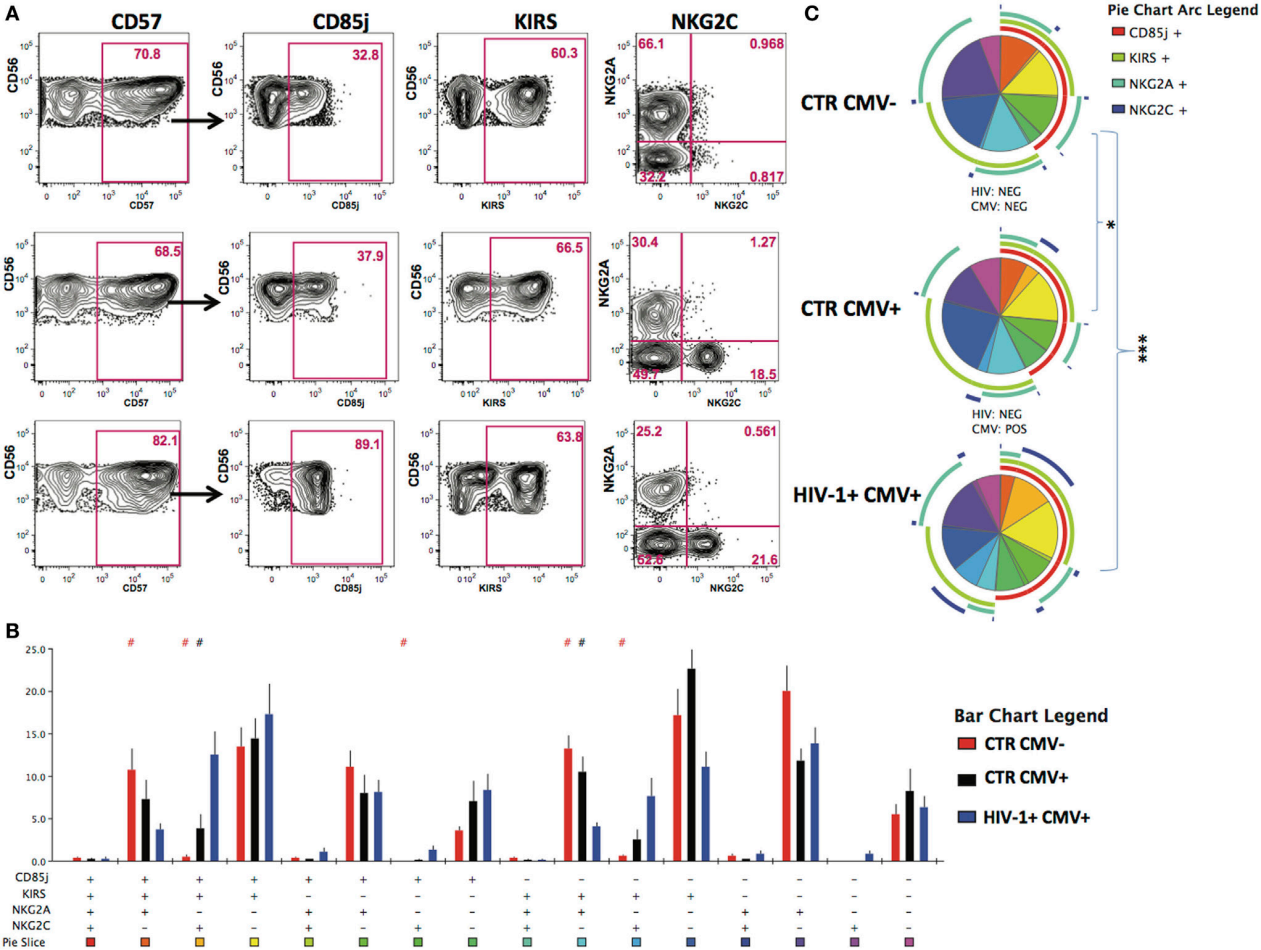
Increased peripheral transition toward a mature CD56dim phenotype in HIV-1 infection. **(A)** Representative contour plots depicting the gating strategy for the expression of CD85j, KIRs, and NKG2A/2C within the CD57-positive CD56dim subset for the three study groups. **(B)** SPICE analysis (bar charts) of combinations of CD85j, KIRs, NKG2A, and NKG2C within the CD57+ fraction of CD56dim natural killer cell subset between HIV-1 seronegative human cytomegalovirus (HCMV) seronegative *n* = 10 (CTR CMV−), HIV-1 seronegative HCMV-seropositive *n* = 10 (CTR CMV+), and HIV-1-infected HCMV-seropositive *n* = 20 (HIV-1+ CMV+) individuals. **(C)** SPICE analysis pie charts for each group. Each colored pie slice corresponds to the different receptor combinations shown. The pie arcs depict individual receptor expression. A permutation test was performed between pie charts (*p < 0.05 and ***p < 0.001). Student’s *t*-test was used to compare samples in SPICE (**^#^***p* < 0.05).

Given that HCMV infection induces a highly differentiated phenotype within the CD56dim subset we assessed the surface expression of NKG2C/A, KIRs (focusing on mainly in inhibitory KIRs using a cocktail of antibodies against KIR2DL1/S5, KIR2DL2/L3/S2, KIR3DL2, and KIR3DL1), CD85j/LIR-1 and the terminal differentiation marker CD57 on this subset in relation to HCMV serostatus in the entire study cohort. HCMV seropositivity in both HIV+ and HIV− subjects was associated with a higher frequency of CD56dim NK cells expressing NKG2C and lower expression of NKG2A compared with HCMV seronegative controls (Figure 1D). A broader range of NKG2C expression was observed in the HIV-1-infected individuals compared with the HIV-1 seronegative HCMV-seropositive controls (range 1.89–81.1 versus 0.263–30.6) likely suggesting higher HCMV burden/reactivation in the former group (31). Higher levels of CD85j were associated with HIV/HCMV co-infection, consistent with reports supporting a role in influencing the long-term responses of CD85j+ NK cells during HCMV dissemination (32). HCMV serostatus did not significantly alter the expression of KIRs (Figure 1D). Expression of CD57 did not differ between the three groups, demonstrating the presence of NK cells at different stages of differentiation (Figure 1D). However, Boolean gating analysis for the expression of NKG2A/C, CD85j, and KIRs within the CD57+ fractions of CD56dim NK cells revealed a higher percentage of NK cells that were CD85j+ KIR+ NKG2C+ and a reduction of the early mature KIR+ NKG2A+ NK cells in the HIV-1-infected individuals compared with both HIV-seronegative control groups; this difference was more pronounced when compared with the HIV-seronegative HCMV-seropositive group (Figures 2A–C). These data are consistent with a more prominent skewing of the NK cell repertoire toward a terminally mature phenotype driven by HCMV co-infection within the HIV-1-infected individuals. This is further supported by downregulation of NCRs (NKp30 and NKp46) in HIV-1-infected individuals, but comparable levels of NKG2D and SLAMF receptors between the groups (Figure S1A in Supplementary Material).

The temporal evolution of the NK cell repertoire was examined in selected individuals (*n* = 5) from whom samples were available during early infection, established viremic infection and at least 1-year post successful antiretroviral-associated viral control. Our analysis revealed that receptor changes evident in early infection persist during chronic disease and in the first year post successful ART (Figures S1B,C in Supplementary Material).

### Gradual Shift in the Functional Responsiveness of the CD56dim NK Cell Subset with Increasing Differentiation

**Figure 3:**
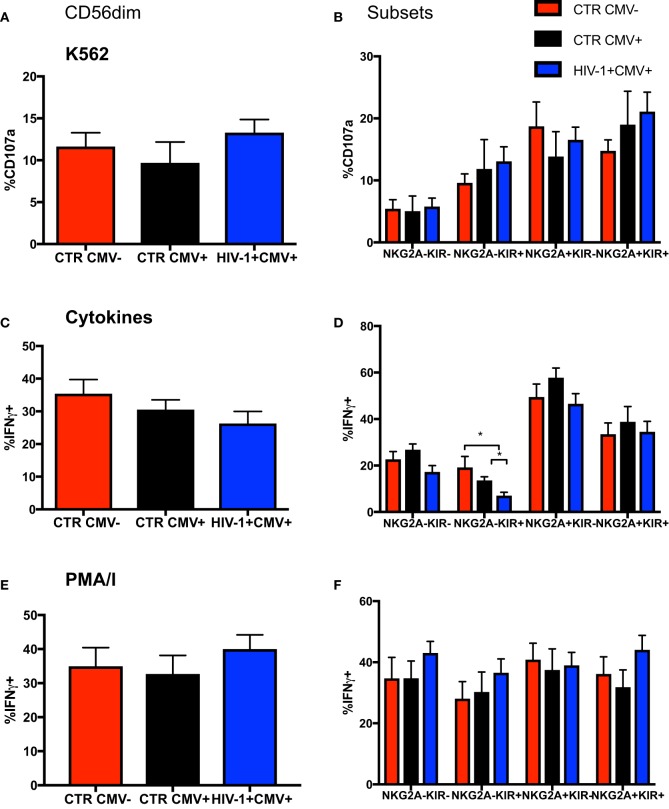
Changes in functionality with differentiation of CD56dim natural killer (NK) cells. Summary bar charts of **(A)** CD107a expression by total CD56dim NK cells and **(B)** subsets thereof distinguished on the basis of the expression of NKG2A and KIRs in the three study groups. **(C)** Summary bar charts showing expression of IFN-γ by total CD56dim NK cells and **(D)** subsets thereof following stimulation with a combination of IL-12/IL-18. **(E)** Summary bar charts of IFN-γ production by total CD56dim NK cells and **(F)** subsets thereof following stimulation with PMA/I. Bars show mean ± SEM. The non-parametric Mann–Whitney *U*-test was used to compare data between groups (*p < 0.05).

Focusing on NKG2A and KIRs as key markers in the dynamic process of terminal differentiation within the CD56dim subset we assessed the ability of CD56dim NK cell subsets at diverse differentiation stages to degranulate following stimulation with K562 (HLA class I devoid) targets. No differences were seen between groups in the ability of CD56dim NK cells and subsets to degranulate on exposure to HLA-I negative targets (Figures 3A,B); the CD56dim NKG2A-KIR− NK cells exhibited significantly lower degranulation against K562 targets cells than the other CD56dim subsets, as previously described (33). Next we evaluated the ability of the CD56dim subset to produce IFN-γ in response to stimulation with IL-12/18. Despite a trend for lower IFN-γ production in the HIV-1-infected individuals, no significant difference was observed in the overall cytokine responsiveness of total CD56dim NK cells between the groups (Figure 3C). This may reflect the relatively small size of the control groups and/or the fact that HIV-1 infection was not extremely advanced (mean days since diagnosis with acute seroconversion = 764.5, mean CD4 = 482) in our study group. However, IL-12 and IL-18 co-stimulation induced significantly less IFN-γ production by the NKG2A-KIR+ subset relative to all other NK cell subsets especially within HCMV-seropositive HIV-1-infected subjects, in whom there is enrichment of more terminally mature CD56dim NK cells (Figure 3D). This is in keeping with the reported reduced expression of cytokine IL-12Rβ2 and IL-18Rα receptors with increasing status of NK cell maturation (34). NK cells did not have an intrinsic defect in cytokine production, as shown by the comparable ability of total CD56dim NK cells and subsets, defined on the basis of NKG2A and KIR expression, to produce IFN-γ in response to phorbol 12-myristate 13-acetate (PMA) and ionomycin (I) stimulation (Figures 3E,F).

### Adaptive CD56dim NK Cells in HIV-1 Infection Are Characterized by Marked Downregulation of PLZF

**Figure 4:**
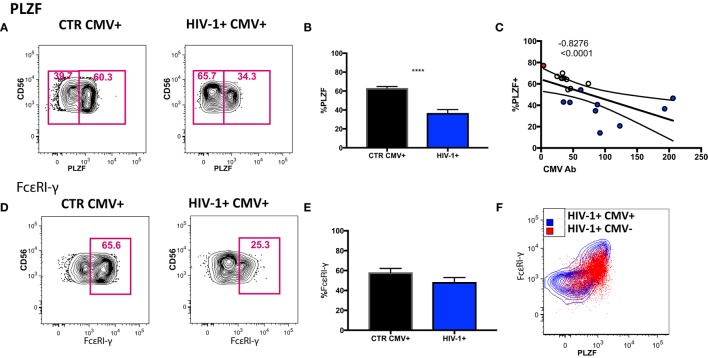
Transcriptional signature of the CD56dim natural killer (NK) population and subsets thereof in human cytomegalovirus (HCMV)-seropositive HIV-infected and -uninfected subjects. **(A)** Representative plots of PLZF expression in CD56dim NK cells from an HIV-1 negative HCMV+ individual and an HIV-1-infected HCMV+ individual and **(B)** summary data from *n* = 10 HIV-1-infected HCMV+ subjects (HIV-1+ CMV+) and *n* = 9 HIV-1 negative HCMV+ controls (CTR CMV+). **(C)** Correlation between PLZF expression levels and available HCMV Ab titers (black circles CTRCMV+, blue filled circles HIV-1+ CMV+, and red filled circle single HIV-1+ CMV−). **(D)** Representative plots of FcεRI-γ expression in CD56dim NK cells from a HIV-1 negative HCMV+ individual and a HIV-1-infected HCMV positive individual and **(E)** summary data. **(F)** Example of PLZF/FcεRI-γ co-expression in a representative HIV-1-infected HCMV+ individual (blue contour) and the HIV-1-infected HCMV− individual (in red). Results are expressed as mean ± SEM (***p < 0.001) by Mann–Whitney test. The non-parametric Spearman test was used for correlation analysis.

To determine to what extent the above phenotypic and functional perturbations reflect the presence of adaptive NK cells within the CD56dim subset in our HIV-1-infected cohort we extended our phenotypic analysis to encompass expression of the transcription factor PLZF, expression of which is reduced in HCMV+ individuals, denoting the presence of adaptive NK cells (15). We detected a stronger downregulation of PLZF expression in the CD56dim subset in the HIV-1-infected HCMV-seropositive individuals compared with HIV-1-seronegative HCMV-seropositive controls (Figures 4A,B). The levels of PLZF expression correlated negatively with serum HCMV Ab titers (Figure 4C). Next we looked at the expression levels of the adaptor protein FcεRI-γ, since a population that lacks FcεRI-γ expression has been found to be strongly associated with HCMV seropositivity and has also been identified in HIV-1 viremic individuals (35, 36). There was no statistically significant difference between the levels of expression of FcεRI-γ in HIV-1-infected HCMV seropositive individuals and HIV-1 seronegative HCMV-seropositive controls (Figures 4D,E) and no significant correlation with CMV Ab levels (correlation *p* = 0.0572 and *r* = −0.4882). Notably the single HCMV− HIV-1-infected individual displayed high expression of PLZF (77.1%) and moderate expression of FcεRI-γ (56.5%) (Figure 4F).

### Phenotypic Profile of PLZF− CD56dim NK Cells in HIV-1 Infection

**Figure 5:**
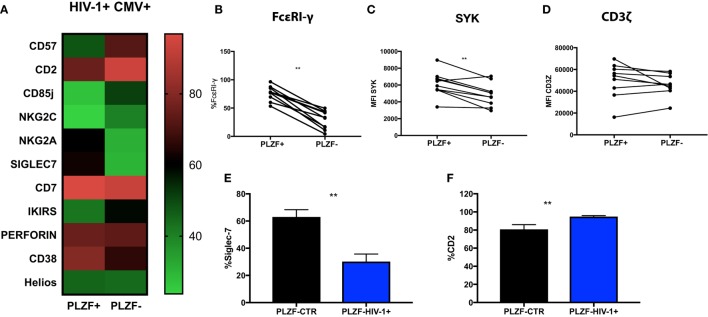
Phenotypic profiles of PLZF− CD56dim natural killer (NK) cells in HIV infection. **(A)** Heat map representation of the mean level of expression (mean + SD) of the receptors shown within the PLZF− and PLZF+ CD56dim NK cell subsets from *n* = 10 HIV-1-infected HCMV+ individuals. Paired data showing proportion of FcεRγ expressed **(B)** and MFI of Syk **(C)** and CD3ζ **(D)** on PLZF− and PLZF+ CD56dim NK cell subsets from *n* = 10 HIV-1-infected HCMV+ individuals. **(E,F)** Bar charts showing proportion of Siglec-7 **(E)** and CD2 **(F)** expressed on PLZF− CD56dim NK cells in HIV-1-uninfected HCMV+ individuals (PLZF-CTR) and HIV-1-infected individuals (PLZF-HIV-1−). Significance determined by the Mann–Whitney test for comparison between groups and the Wilcoxon signed rank test for paired data (**p < 0.01).

To examine the nature of the PLZF− subset in relationship to the PLZF+ subpopulation, we performed a detailed evaluation of their phenotypic attributes. PLZF− CD56dim NK cells in HIV-1-infected individuals exhibited coordinated changes in the expression of a number of molecules, some of which have been previously associated with the differentiation status and adaptive-like phenotype of NK cells (Figure 5A). We observed significantly increased expression of CD57 (*p* = 0.020), CD85j (*p* = 0.0010), KIRs (*p* = 0.0312), and CD2 (*p* = 0.0010) within the PLZF− subset relative to the PLZF+ subset. By contrast there was a significant under-representation of NKG2A (*p* = 0.0020), CD7 (*p* = 0.0010), Siglec-7 (*p* = 0.0010), and CD38 (*p* = 0.0371) within the PLZF− subset, in keeping with a more mature and adaptive-like phenotype. A non-statistically significant trend for higher expression of NKG2C in the PLZF− subset (*p* = 0.0557) was seen, suggesting that not all NKG2C+ NK cells possess an adaptive signature. No difference was detected in the intracellular levels of perforin (*p* = 0.6250) between the two subsets. Equally there was no significant decrease in the levels of Helios in the PLZF− versus PLZF+ subset (*p* = 0.7002), consistent with observations that Helios does not appear to be uniformly downregulated in human adaptive NK cells (15).

PLZF can initiate changes in DNA methylation and is known to interact with the promoter regions of FcεRI-γ and Syk. Accordingly FcεRI-γ− and Syk− NK cell subsets were enriched within the PLZF− NK cell population (*p* = 0.0010 and *p* = 0.0059, respectively) (Figures 5B,C). CD3ζ levels were comparable between the two subsets, in line with reports demonstrating that PLZF does not bind the promoter of the gene encoding CD3ζ (Figure 5D) (15). Similar changes were observed in the two subsets from healthy HIV-1 seronegative HCMV-seropositive individuals (data not shown). A more striking downregulation of Siglec-7 and upregulation of CD2 was, however, noted in total CD56dim and NK cell subsets in HIV-1-infected individuals compared with HIV-1-uninfected HCMV-seropositive controls (*p* < 0.0001 and *p* = 0.0074, respectively, data not shown) and within the PLZF− fractions (Figures 5E,F). The expression of CD2 in the PLZF− fraction of NK cells correlated positively with CMV Ab levels (*r* = 0.7053, *p* = 0.0007). No correlation was observed between expression of Siglec-7 and CMV Ab titers (*r* = −0.16, *p* = 0.5261). Interestingly, the single HIV-1-infected HCMV seronegative patient had decreased expression of Siglec-7 (total expression on CD56dim cells 33% versus 39.82% mean expression in HIV-1+ HCMV+ individuals) and the lowest level of expression of CD2 (71.5% versus mean expression 86.4% within the HIV-1+ HCMV+ individuals).

A subpopulation of PD1+ NK cells, comprised of fully mature NK cells, has been recently described in HCMV+ individuals (37). We detected a modest increase in the levels of PD1 expression within the PLZF− subset of NK cells in HIV-1-infected individuals (Figures S2A,B in Supplementary Material). The two individuals with the highest levels of PD1 expression had a lower CD4 count (mean = 190), higher HIV-1 viral load (>100,000 copies/mL) and a more prolonged course of disease (mean = 1,535 days since seroconversion).

### Adaptation of the CD56dim NK Cell Compartment to HCMV Results in Stable Imprints during the Course of HIV-1 Infection

**Figure 6:**
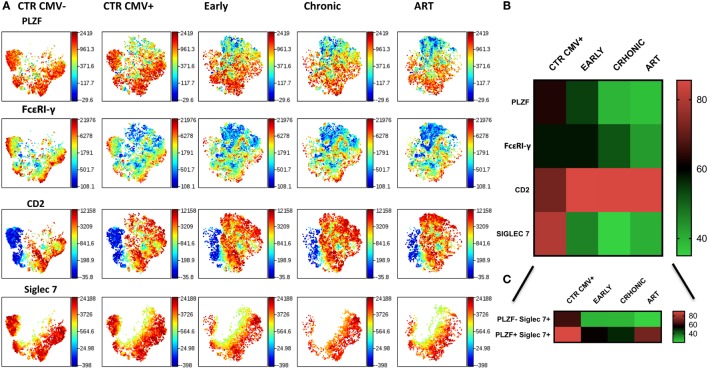
Stability of CD56dim transcriptional signature during HIV-1 infection. **(A)** viSNE analysis of multiparametric data was performed on CD56dim natural killer (NK) cells from five compiled HIV-1-infected donors during early and chronic infection and post-antiretroviral (ART). Representative data from a HIV-1-negative HCMV-negative (CTR CMV−) and composite data from 9 HIV-1-negative HCMV-positive control individuals (CTR CMV+) are shown for comparison. For better visualization of the intensity of expression of Siglec-7, the Siglec-7+ population was selected. Each point in the viSNE map represents an individual cell. Colors depict intensity of protein expression. **(B)** Heat maps of levels of expression (mean + SD) of PLZF, FcεRI-γ, CD2, and Siglec-7 in *n* = 5 HIV-1-infected HCMV+ individuals and *n* = 9 HIV-1-uninfected HCMV+ controls. **(C)** Heat map representation of the levels of expression of Siglec-7 within the PLZF− and PLZF+ fractions of NK cells.

To examine the longitudinal changes in NK cell subsets with an adaptive signature in HIV-1 infection we compared the expression of Siglec-7, CD2, FcεRI-γ, and PLZF in CD56dim NK cells during early, established infection and post-ART in 5 individuals. In addition to flow cytometric analysis we employed non-linear dimensionality reduction using t-SNE (26). This enabled us to visualize high-dimensional data in two-dimensional representations and circumvent any bias introduced by manual gating. Analysis revealed clear clusters of cells sharing low expression of Siglec-7, FcεRI-γ, and PLZF and high CD2 expression (Figure 6A). Importantly, these changes were apparent during early infection, were more pronounced during established chronic infection, and were maintained after successful ART (Figure 6B), with the exception of Siglec-7, which demonstrated some recovery of expression in the PLZF+ subset (Figure 6C). A non-significant trend toward even lower levels of FcεRI-γ was observed during treatment (Figure 6B). PLZF can initiate changes in DNA methylation and interacts with the promoter regions of FcεRI-γ and several other target genes (15), explaining the stability of the adaptive signature after treatment. This is further supported by a recent report indicating that 2 years of successful ART does not reverse CD56dim FcεRγ− NK cell expansion (36).

### Functional Correlates of CD56dim NK Cells with an Adaptive Signature in HIV-1 Infection

**Figure 7:**
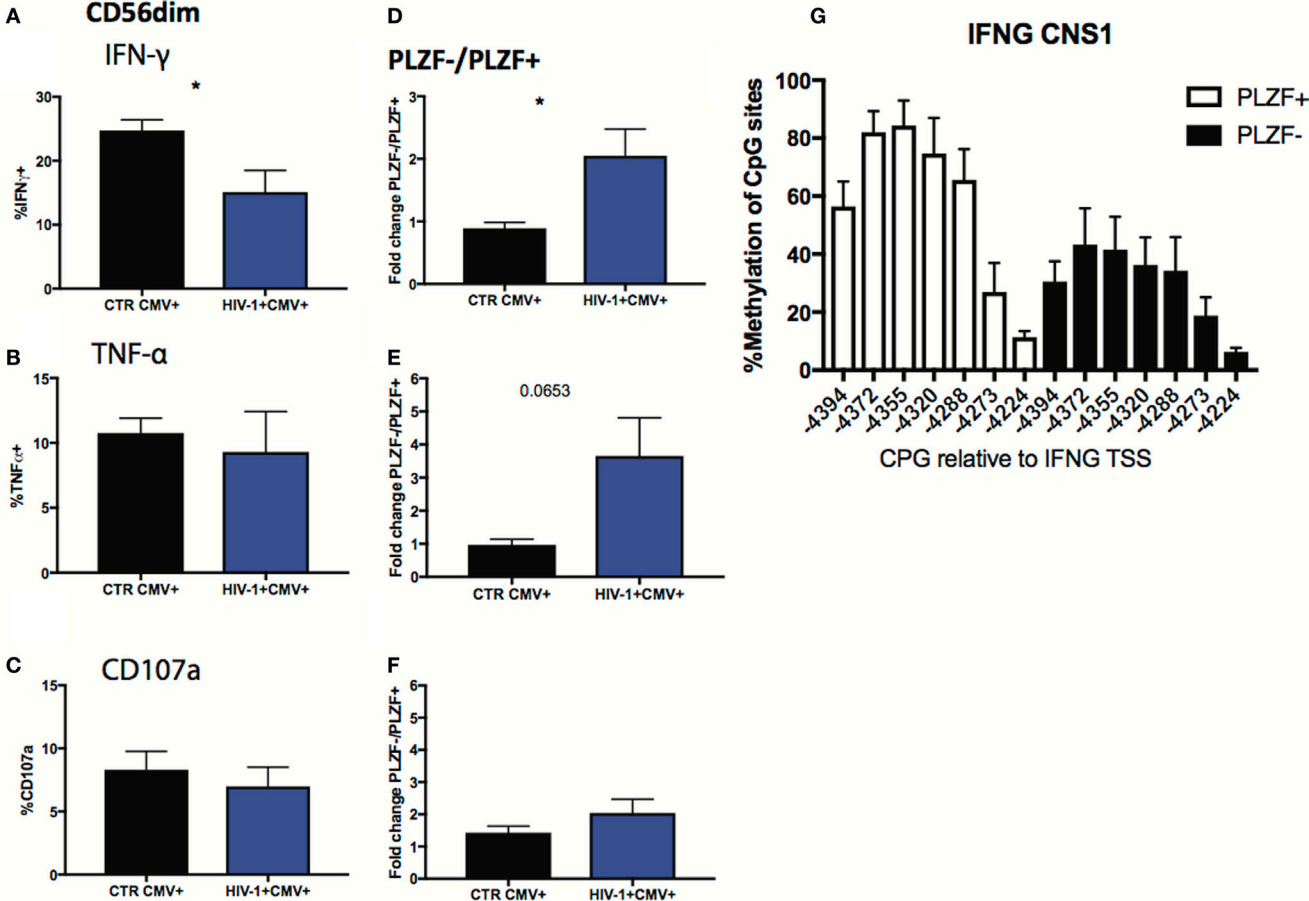
Functional responses of CD56dim subsets to CD16 triggering. **(A–C)** Summary bar charts of IFN-γ, TNF-α, and CD107a production by CD56dim natural killer (NK) cells from human cytomegalovirus (HCMV)-seropositive HIV-1-uninfected and -infected individuals. **(D–F)** Summary bar charts of fold change in IFN-γ, TNF-α, and CD107a production between PLZF−/PLZF+ NK cells in HCMV-seropositive HIV-1-uninfected *n* = 9 and -infected individuals *n* = 10. Bars mean ± SEM. Significance determined by the Mann–Whitney test for comparison between groups, *p < 0.05. **(G)** Bar graphs showing the mean percentage ± SD of methylation detected at each individual site of the *IFNG* CNS1 locus in PLZF+ (white bars) and PLZF− (black bars) NK cell subsets from *n* = 3 HIV-1/HCMV+ infected individuals.

Finally, we sought to investigate the functional consequences of the observed alterations in the NK cell signaling and transcription factors. Given the importance of antibody-dependent cellular cytotoxicity (ADCC) in the control of HCMV, we analyzed the Fc receptor-dependent activation of adaptive NK cells in response to plate bound anti-CD16 stimulation. The percentage of total CD56dim NK cells from HIV-1-infected individuals producing IFN-γ in response to stimulation was significantly lower than that in HIV-1 seronegative HCMV-seropositive individuals (Figure 7A), with less marked effects being observed on TNF-α production and degranulation (Figures 7B,C). As a potential mechanism explaining the overall reduced proportion of NK cells able to produce IFN-γ we determined the levels of expression of CD16 (Figures S3A,B in Supplementary Material), which correlated positively with IFN-γ production (*r* = 0.5573, *p* = 0.0132), suggesting that CD16 downregulation or shedding during chronic HIV-1 infection may impair the overall functional ability of NK cells. Despite the overall reduction in IFN-γ production, the fold change in the capacity of PLZF−/PLZF+ subsets to produce IFN-γ was greater in the HIV-1-infected individuals compared with HCMV+ HIV-1− controls (Figure 7D) in whom canonical PLZF+ NK cells displayed higher production of IFN-γ after cross-linking (Figure S3C in Supplementary Material). A similar trend was observed for TNF-α production, although the degranulation capacity of PLZF−/PLZF+ subsets did not differ (Figures 7E,F). The enhanced ability of the PLZF− subset, which is enriched in the HIV-1-infected group, to produce IFN-γ is in keeping with a more hypomethylated and hence accessible region of the CNS1 region of the IFN-γ locus in the adaptive PLZF− NK cells (Figure 7G).

## Discussion

This study demonstrates that viremic HIV-1-infected patients manifest phenotypic and functional perturbations in their NK cell compartment consistent with an adaptive reconfiguration driven in part by underlying co-infection with HCMV, an almost ubiquitous pathogen in HIV-1-infected cohorts and a recognized cofactor associated with ongoing immune activation. The complex interplay between HCMV and HIV-1 likely leads to the establishment of a predominantly adaptive-like CD56dim NK cell profile further delineated by a stronger downregulation of the transcription factor PLZF (thought to mark adaptive NK cells in humans (15)), compared with HIV-1-uninfected HCMV-seropositive individuals. The PLZF− NK cell subset in HIV-1 infection was found to be enriched in markers denoting maturation, and to have an enhanced capacity for IFN-γ production.

The factors accelerating the acquisition of a fully mature CD56dim CD57+ KIR+ NKG2C+ CD85j+ NKG2A− phenotype during HIV-1 infection in comparison with HIV-1-HCMV− individuals and driving a greater loss of PLZF expression in HIV-1-infected patients compared with HIV-1-uninfected HCMV-seropositive controls remain unclear. The potential role of HCMV as a driving force behind the acquisition of an adaptive NK phenotype during HIV infection is suggested by the fact that PLZF levels correlated in a negative fashion with HCMV Ab titers. The PLZF− CD56dim subset in HIV-1-infected individuals was distinguished by more pronounced upregulation of CD2 and downregulation of Siglec-7 compared with HIV-1-uninfected HCMV-seropositive individuals. CD2, a co-activating receptor, synergizes with CD16 to boost cytokine production in adaptive NK cells (16). CD2 expression also correlated positively with CMV Ab levels, raising the possibility that CD2, through its recognition of its ligand CD58 on HCMV-infected cells, may influence the shaping of adaptive NK cells by providing a co-stimulatory signal. Siglec-7, an inhibitory receptor expressed constitutively on all NK cell subsets, is thought to participate in the regulation of cell function and survival (38). In addition to the global downregulation of Siglec-7 on CD56dim NK cells previously reported both in HIV-1 infection (39, 40) and in HSCT patients reactivating HCMV (41), we detected a more profound loss of Siglec-7 within the PLZF− subset in HIV-1 infection. It has been suggested that cumulative exposure to high levels of HIV-1 viral replication may induce Siglec-7 shedding from the surface of NK cells (42). More recently downregulation of Siglec-7 on activated PBMCs was reported under diabetogenic and inflammatory conditions (43). Thus systemic inflammation and metabolic abnormalities during HIV-1 infection could modulate the expression of this receptor. Differentiation of adaptive NK cells is also driven by inflammation in animal models, and NK cells can display memory-like responses secondary to cytokines (44). Together, these data suggest that a combination of ongoing immune activation, higher infectious burdens including HCMV and progressive immune dysregulation could contribute to the higher expansions of NK cells with adaptive features in HIV-1-infected individuals. Importantly these phenotypic changes persist during ART especially within the PLZF− subset, which may reflect stable epigenetic modifications and the continuous influence of ongoing immune activation despite ART.

The functional capacities of distinct NK cell subsets in HIV-1 infection are consistent with changes observed with increased differentiation (33). The enhanced capacity of the PLZF− NK cell subset for IFN-γ production compared with conventional PLZF+ NK cells in HIV-1 infection, and in comparison with HIV-1 negative HCMV-seropositive controls, is reflective of the greater magnitude of PLZF− NK cells, and suggests a more restricted specificity toward CD16 triggering and cytokine production. The increased *IFNG* CNS1 accessibility could provide a molecular mechanism underlying more potent IFN-γ production following engagement of CD16. Moreover in PLZF− adaptive NK cells, which lack FcεRI-γ, CD16 stimulation could mediate increased downstream signaling through association with CD3ζ homodimers containing a total of six ITAMs (CD3ζ was expressed at comparable levels between adaptive and conventional NK cell subsets). FcεRI-γ− NK cells isolated from HIV-1+ individuals have been shown to respond robustly when stimulated with HIV peptides in the presence of heterologous HIV+ serum (35). The overall reduction in the activity of CD56dim NK cells following CD16 triggering in our HIV-1-infected individuals was associated with differences in *ex vivo* CD16 expression levels, in keeping with previous reports of impaired ADCC function with progressive disease (45). This could be attributable to elevated MMP expression and activity during chronic HIV-1 infection (45). Interestingly in this small cohort we did not observe a significant reduction in the anti-CD16 degranulation response in the HIV-1-infected individuals. It is plausible that different CD16 signaling thresholds exist for degranulation versus cytokine production (46) along with additional regulatory mechanisms. Nonetheless the identification of a subset with retained ability to respond to Fc-triggering in HIV-1 infection has potentially important implications for the design of therapeutic vaccines aimed at generating ADCC-promoting antibody responses. In future, it will therefore important to examine the size of adaptive NK cell populations in larger, demographically diverse cohorts and gain further insight into the role of signaling *via* CD16 and HIV-1 antibody-dependent NK cell activation and cytotoxicity as putative mechanisms for viral control (47).

While the expansion of adaptive NK cells may serve a role in protective immunity against HCMV, important questions remain as to their role in conferring protection against HIV-1 acquisition and how their presence impacts on HIV-1 control following infection. A beneficial role for adaptive NK cells has been suggested in both heterologous infection (20) and leukemia (48). We were not able to explore whether PLZF− NK cells exhibited an enhanced ability to kill HIV-infected cells due to insufficient sample availability. Nonetheless mature or expanded NKG2C+ NKG2A− NK cells have been associated with a better early response to ART, low HIV viral setpoint and superior anti-HIV capacity (49, 50). The converse observations of positive and negative correlations between NKG2C+ and NKG2A+ NK cells, respectively, and HIV-1 viral load during chronic infection (51) may be explained by evolving dysregulation and NK exhaustion during chronic infection, in line with which we observed a modest increase of PD1 expression confined to the PLZF− subsets in individuals with more advanced HIV-1 disease.

Our findings reconcile some of the discrepancies in the published literature, demonstrating the clear footprint of HCMV co-infection on NK cell phenotype during HIV-1 infection. However, a number of other factors, including the degree of immunosuppression and underlying level of pro-inflammatory cytokines during HIV-1 infection, could further shape the NK cell repertoire and accentuate the magnitude of these changes. The possibility for careful manipulation of specific NK cell subsets with unique properties to achieve better HIV-1 control is an exciting avenue for further investigation. However, these attempts need to be tempered with the potential reduced tonic signaling from activating receptors in adaptive NK cells, which could influence their immunoregulatory and tumor surveillance role.

## Ethics Statement

This study was approved by the National Health Service Camden and Islington local research ethics committee (LREC 98/60) and carried out in accordance with their recommendations with written informed consent from all subjects. All subjects gave written informed consent in accordance with the Declaration of Helsinki.

## Author Contributions

DP performed experiments; contributed to study design, acquisition of data, analysis, and drafting of the manuscript; IP-P performed experiments and contributed to acquisition of data; PP and IW contributed to study design and data interpretation; MM and PB contributed to study design, data interpretation, and editing the manuscript.

## Conflict of Interest Statement

The authors declare that the research was conducted in the absence of any commercial or financial relationships that could be construed as a potential conflict of interest.
