# Supplementary material for: Adaptive Reconfiguration of Natural Killer Cells in HIV-1 Infection
Source: Front Immunol. 2018 Mar 16;9:474. doi: 10.3389/fimmu.2018.00474 (PMC5864861; doi:10.3389/fimmu.2018.00474)
Supplement: Supplementary file 1 [file table_1.docx]

**Table S1. Cohort characteristics.**

| **Cohort Characteristics** | **Days since diagnosis with acute seroconversion** | | **Age** | **VL copies/mL** | **CD4+ cells/uL** |
| --- | --- | --- | --- | --- | --- |
| **Viraemic HIV-1+**  Sex: Male; Caucasian  CMV Seropositive n=20  CMV Seronegative n=1 | Mean: 764.5 (306-1786  1329 | Mean: 40 (26-57)  36 | | Mean: 87111 (1400-430300)  336300 | Mean: 482(190-880)  310 |
| **HIV-1 Seronegative Controls**  Sex: Male; Caucasian | NA |  | | NA | NA |
| CMV Seropositive n=10 |  | Mean: 42 (28-55) | |  |  |
| CMV Seronegative n=10 |  | Mean: 35.4 (25-45) | |  |  |

*NA= not applicable*
