# Supplementary material for: Adaptive Reconfiguration of Natural Killer Cells in HIV-1 Infection
Source: Front Immunol. 2018 Mar 16;9:474. doi: 10.3389/fimmu.2018.00474 (PMC5864861; doi:10.3389/fimmu.2018.00474)
Supplement: Supplementary file 2 [file table_2.docx]

**Table S2. Cohort Characteristics of longitudinally sampled subjects.**

| **Cohort Characteristics**  **Paired samples**  **HIV-1+**  **HCMV Seropositive** | **Days since diagnosis with acute seroconversion (mean)** | **VL copies/mL**  **(mean)** | **CD4+ cells/uL**  **(mean)** |
| --- | --- | --- | --- |
| Early n=5 | 36.6 | 174660 | 660 |
| Chronic n=5 | 905.375 | 68850 | 446.25 |
| ART n=5 | >12 months of ART | <50 | 505 |
