# Supplementary material for: Adaptive Reconfiguration of Natural Killer Cells in HIV-1 Infection
Source: Front Immunol. 2018 Mar 16;9:474. doi: 10.3389/fimmu.2018.00474 (PMC5864861; doi:10.3389/fimmu.2018.00474)
Supplement: Supplementary file 3 [file image_1.PDF]

Figure S1. NKR expression on CD56dim and evolution of expression during the course of HIV-1 infection

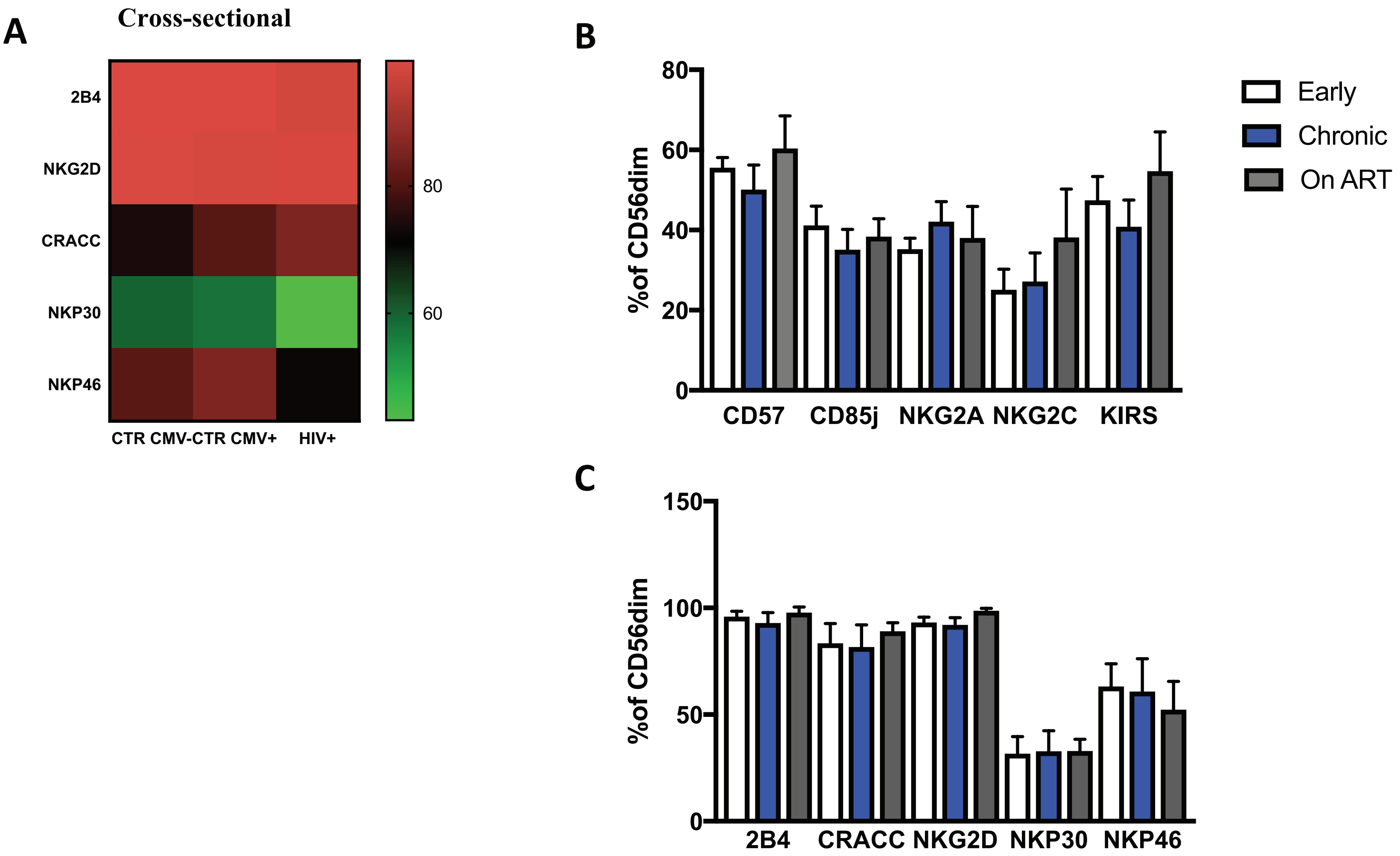

Figure S1. NKR expression on CD56dim NK cells, and evolution of expression during the course of HIV-1 infection.
(A) Heat map representation of the expression (mean expression and SD) for the individual receptors within the 3 groups studied.
(B) and (C) Summary paired data for the ex vivo expression of the receptors shown on CD56dim NK cells from n=5 HIV-1 infected individuals sampled during early and chronic untreated infection and after 12 months of ART.
