# Supplementary material for: Adaptive Reconfiguration of Natural Killer Cells in HIV-1 Infection
Source: Front Immunol. 2018 Mar 16;9:474. doi: 10.3389/fimmu.2018.00474 (PMC5864861; doi:10.3389/fimmu.2018.00474)
Supplement: Supplementary file 4 [file image_2.PDF]

Figure S2. Higher PD1 expression within PLZF- CD56dim NK cells in HIV-1 infection

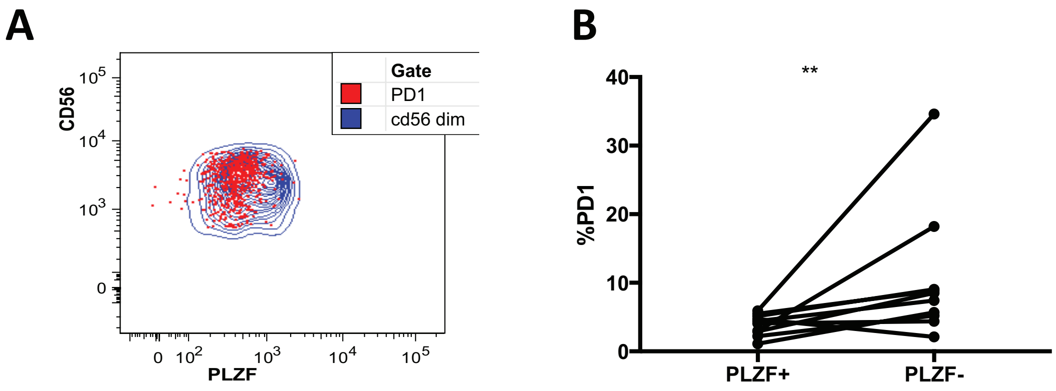

Figure S2. Higher PD1 expression within PLZF- CD56dim NK cells in HIV-1-infected individuals.
(A) Representative plot from a HIV-1 infected HCMV+ individual showing localization of PD1 expression (in red) within the CD56dim PLZF- subset. (B) Summary paired data showing levels of expression of PD1 between PLZF+ and PLZF- subsets in HIV-1 infected individuals. Significance determined by the Wilcoxon signed rank test for paired data, $^{**}p<0.01$.
