# Supplementary material for: Adaptive Reconfiguration of Natural Killer Cells in HIV-1 Infection
Source: Front Immunol. 2018 Mar 16;9:474. doi: 10.3389/fimmu.2018.00474 (PMC5864861; doi:10.3389/fimmu.2018.00474)
Supplement: Supplementary file 5 [file image_3.PDF]

Figure S3. CD16 expression and functional response to CD16 crosslinking within NK subsets

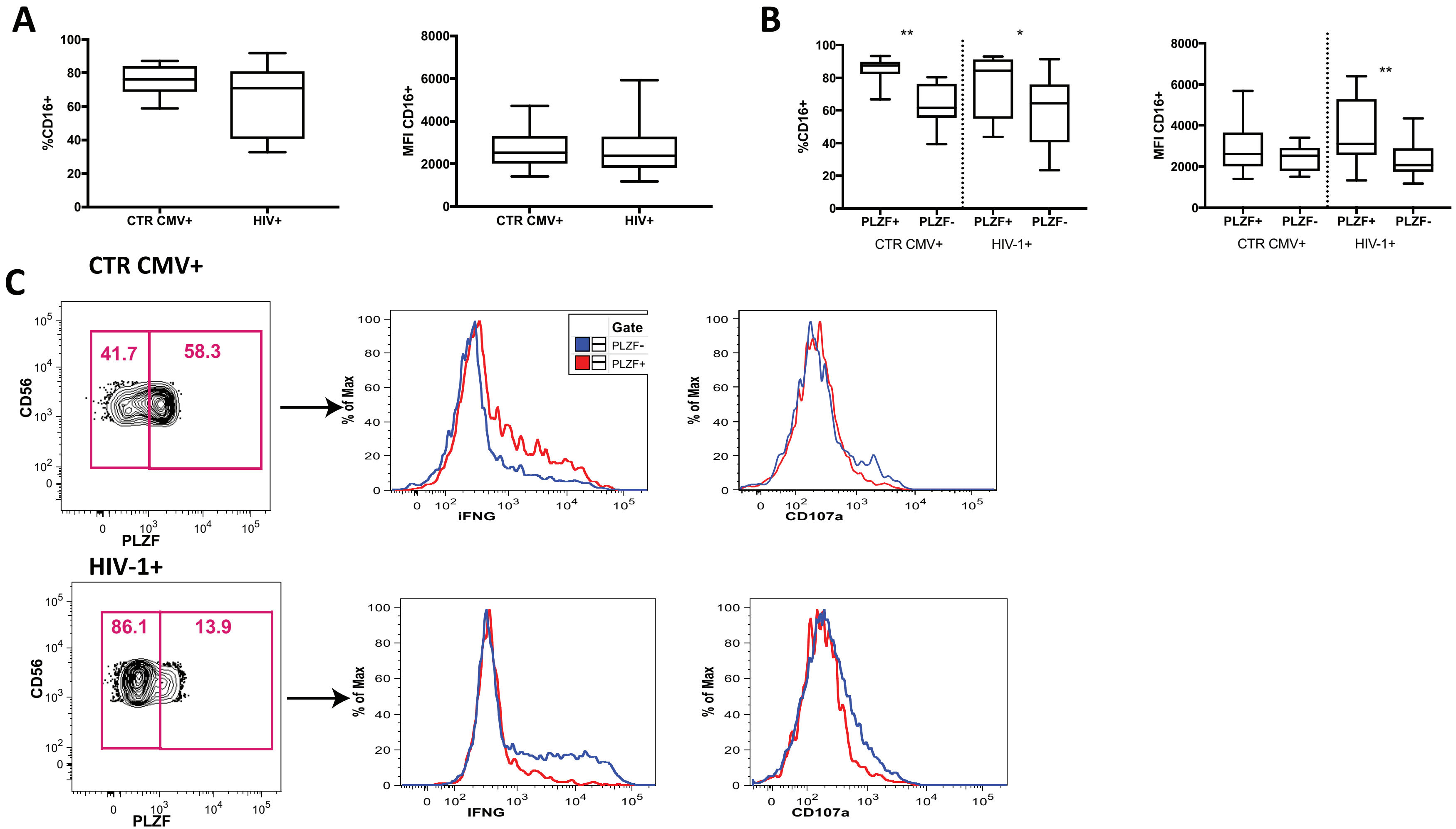

Figure S3. CD16 expression and functional response to CD16 crosslinking within NK subsets.
(A) Summary box plots of the percentage of CD16 and MFI (gated on CD16+ cells) within the CD56dim NK cells in CMV+ seropositve controls and HIV-1 infected individuals. **(B)** Box plots of CD16 percentage and MFI wihtin the PLZF+ and PLZF- fractions from the two study groups. Box-and-whisker plots show the median, quartiles and range. *P<.05, **P<.01**(C)** Representative contour plots from a control CMV+ HIV-1 seronegative individual and an HIV-1 infected subject showing gating for PLZF+ and PLZF- CD56dim subsets and histograms depicting IFN-γ and CD107a expression from each subset PLZF+ (in red) and PLZF- (in blue).
